# Seasonal variation in environmental DNA detection in sediment and water samples

**DOI:** 10.1371/journal.pone.0191737

**Published:** 2018-01-19

**Authors:** Andrew S. Buxton, Jim J. Groombridge, Richard A. Griffiths

**Affiliations:** Durrell Institute for Conservation and Ecology, School of Anthropology and Conservation, University of Kent, Marlowe Building, Canterbury, Kent, United Kingdom; University of Hyogo, JAPAN

## Abstract

The use of aquatic environmental DNA (eDNA) to detect the presence of species depends on the seasonal activity of the species in the sampled habitat. eDNA may persist in sediments for longer than it does in water, and analysing sediment could potentially extend the seasonal window for species assessment. Using the great crested newt as a model, we compare how detection probability changes across the seasons in eDNA samples collected from both pond water and pond sediments. Detection of both aquatic and sedimentary eDNA varied through the year, peaking in the summer (July), with its lowest point in the winter (January): in all seasons, detection probability of eDNA from water exceeded that from sediment. Detection probability of eDNA also varied between study areas, and according to great crested newt habitat suitability and sediment type. As aquatic and sedimentary eDNA show the same seasonal fluctuations, the patterns observed in both sample types likely reflect current or recent presence of the target species. However, given the low detection probabilities found in the autumn and winter we would not recommend using either aquatic or sedimentary eDNA for year-round sampling without further refinement and testing of the methods.

## 1. Introduction

The advent of aquatic environmental DNA (eDNA) protocols for surveying aquatic organisms has revolutionised the assessment of both protected and invasive species. Extra-organismal DNA is collected as part of a sample of environmental material and isolated in a laboratory to identify the recent presence of a species [1–4]. However, as with all survey methods, sampling aquatic eDNA is limited to time periods when the species is active and in its aquatic phase. DNA bound to sediments has been found to persist much longer [5], and therefore may be an appropriate source of DNA to allow the detection of a species outside its active period.

Animals constantly shed DNA into their environment through the expulsion of waste products, skin secretions, sloughing of skin cells, release of reproductive cells (eggs and sperm), through the decay of dead individuals and through many other processes [6–8]. This organic material becomes suspended in the water column [2]. The persistence of aquatic eDNA depends on a range of factors and is highly variable [9–11]. eDNA is broken down through both biotic and abiotic processes [4,10,12–16]. eDNA in marine or lotic environments can be transported out of the system it was released in and diluted to undetectable levels [9,12]. Additionally, eDNA becomes undetectable by settling out of the suspension through vertical transport and incorporation into sediment [5]. This process may result in progressive accumulation of eDNA in the sediment [17]. The rate at which particles settle out and therefore the amount of eDNA suspended within the water column is related to particle size [18]. Turner *et al*. [16] found that although the highest amounts of total eDNA pass through 0.2 μm filters, 71% of targeted carp eDNA was trapped by 1 μm filter membranes. Particles greater than 1 μm therefore settle out of natural waters [19] and accumulate in the sediment [16]. Consequently, sediment may be a valuable but as yet largely untested source of eDNA.

Within the sediment, extracellular DNA can bind to the mineral particles and humic compounds [20–22], with the capacity varying with sediment characteristics [13,23]. Long-term persistence of the DNA molecules is therefore predominantly due to bound DNA molecules being protected from degradation [22,24–27]. Consequently, DNA has the potential to persist in the sediment for a short time or for thousands of years [7,28–35] depending on these conditions.

The isolation of DNA from sediment was developed with microbial DNA [36]. The field of ancient DNA has subsequently emerged using the same principles to isolate DNA from terrestrial and aquatic sediments [28,31,32,37]. However, techniques to isolate contemporary DNA from soils or aquatic sediments have emerged only relatively recently. The potentially extensive persistence of DNA bound to sediments is very valuable for analysis of ancient DNA, but it may be difficult to identify when the target species was last present. In experimental conditions, big headed Asian carp (*Hypophthalmichthys* spp.) eDNA has been found to persist in sediments for longer than four months and to be more concentrated in the sediment than the water column [5].

With either direct field observation or aquatic eDNA surveys, the short survey season available for semi-aquatic species such as amphibians can reduce the application of the method. For protected species, missing the effective survey window can lead to false negatives and poorly informed conservation decision-making, which has potential economic implications. Reliable year-round detection methods that can detect the recent presence of a species therefore have great benefits. Year-round detection using aquatic eDNA has been proposed with great crested newts (*Triturus cristatus*) [38], a semi-aquatic amphibian with which eDNA has become rapidly adopted as a survey protocol [11,38–41]. Simple positive or negative results from a single eDNA sample are being used for distribution assessments of the species and to inform mitigation of development impacts on newt habitat [42]. However, the reliability of this in different seasons has not been assessed. We use great crested newts as a model species to examine the reliability of eDNA sampling in different seasons. In addition we develop a method to extract eDNA from pond sediments and assess how the probability of detection changes seasonally and how it compares to aquatic eDNA samples. We discuss whether eDNA from pond sediment could be used to allow year-round detection for a semi-aquatic species.

## 2. Methods

### 2.1. Study areas

Eighteen ponds in three study areas in south and south-east England and known to support great crested newts were chosen. These comprised eight ponds at Little Wittenham in Oxfordshire, a designated Special Area of Conservation (SAC) for great crested newts; and two study areas in Essex, both created as mitigation habitat for local development projects containing translocated individuals, one at Wickford (six ponds) and one at Stanford-le-Hope (four ponds). An additional pond located in an isolated position inaccessible to great crested newt colonisation on an island in the centre of Canterbury City, was used as a negative control and a second negative control pond which had yet to establish vegetation and had no record of great crested newts was located near the Stanford-le-Hope population.

### 2.2. Visual surveys and Habitat Suitability Index

A combination of torch-light surveys, aquatic funnel traps and visual searches for eggs were used to confirm the presence of great crested newts in each pond [43]. A well-established Habitat Suitability Index (HSI) assessment exists for great crested newts and was calculated for each pond [44]. The HSI is a measure of the suitability of a pond and associated habitat for the target species [44]. Ten habitat variables are recorded in the field, comprising geographic location (SI_1_), pond area (SI_2_), frequency of pond drying (SI_3_), water quality (SI_4_), pond shading (SI_5_), waterfowl presence (SI_6_), fish presence (SI_7_), pond density in the immediate landscape (SI_8_), terrestrial habitat quality (SI_9_) and macrophyte cover (SI_10_), and are each scored between 0.01 and 1.0. The final HSI index is calculated as the geometric mean of the variables using the equation [44]:

$$\text{HSI}={{(\text{SI}}_{1}*{\text{SI}}_{2}*{\text{SI}}_{3}*{\text{SI}}_{4}*{\text{SI}}_{5}*{\text{SI}}_{6}*{\text{SI}}_{7}*{\text{SI}}_{8}*{\text{SI}}_{9}*{\text{SI}}_{10})}^{1/10}$$

The index gives a broad indication of the quality of the habitat for great crested newts on a numerical scale of 0 (unsuitable habitat) to 1 (optimal habitat) [45].

### 2.3. Sample collection

All equipment was sterilised using a 10% bleach solution and/or UV light. Before sampling the sediment, an aquatic eDNA sample was collected from the undisturbed water column. The aquatic eDNA sampling followed a precipitation in ethanol method described in Biggs *et al*. [39], and replicated the commonly used protocol for commercial great crested newt eDNA sampling in the UK. To allow a single representative sample of sediment to be collected from a pond, ten subsamples were collected from the accessible pond perimeter at evenly spaced intervals and combined. Using shoulder length disposable gloves to avoid contamination, a 60 mL scoop of the surface of the pond sediment was collected in a polypropylene collection pot from the ten sampling locations. Any pond water collected as part of the sampling process was then drained off and the sediment transferred to a 1000 mL wide-mouth plastic bottle. 250 mL of double distilled water was then added to the sample, and the bottle shaken vigorously for 60 seconds to suspend the sediment within the distilled water. Fifteen mL of this solution was immediately subsampled and added to a 50 mL centrifuge tube containing 33 mL of absolute ethanol and 1.5 mL of 3 M sodium acetate solution to preserve the sample. The remainder of the distilled water sediment suspension was retained for sediment texture analysis.

Both aquatic and sedimentary eDNA samples were transported on the day of collection to the laboratory at the University of Kent and stored at -20°C until extraction. Samples were collected from ponds at approximately three monthly intervals in April, July and October 2016 and January 2017 to cover the four seasons. If ponds were dry and an aquatic eDNA sample could not be collected then aquatic eDNA was considered to be negative in the analysis.

### 2.4. eDNA extraction

Extraction of the aquatic eDNA sample followed the same modified Qiagen^®^ DNeasy^®^ blood and tissue extraction kit protocol used by Biggs *et al*. [39]. Extraction of sedimentary eDNA samples followed modified Qiagen^®^ QIAamp^®^ DNA Stool Mini Kit protocol [46]. The 50 mL centrifuge tube containing the ethanol preservative with the suspension of pond sediment and distilled water was removed from the freezer and shaken vigorously to homogenise the sample. The sample was then centrifuged at 8500 rpm for 30 minutes to separate the sediment from the preservative, the supernatant was carefully poured off and discarded. Sediment was removed from the centrifuge tube, placed on a sterile Petri-dish, and then stirred to mix once again. Half of one milliliter of sediment was then transferred to a 2 mL micro-centrifuge tube. Extraction continued as per Chaves *et al*. [46] and is outlined in supporting information S1 Methods. Both aquatic and sedimentary eDNA extracts were stored at -20°C until qPCR could be undertaken.

### 2.5. eDNA qPCR and IPC

Quantitative real-time PCR was undertaken on all samples following the assay and PCR conditions in Biggs *et al*. [39], with PCR primers TCCBL and TCCBR as well as minor groove binding probe TCCB from Thomsen *et al*. [47]. The primers, assay and PCR conditions are outlined in supporting information S1 Methods. Each sample was repeated eight times and run in parallel with both positive and negative controls. All samples were checked for PCR inhibition using TaqMan^®^ Exogenous Internal Positive Control Reagents (Applied Biosystems^™^), following manufacturer’s instructions, with TaqMan^®^ Environmental Master Mix 2.0 (Applied Biosystems^™^). Samples were identified as inhibited if the IPC failed to amplify or late amplification (amplification outside 1 qPCR cycle from the qPCR negative control samples) was observed within the internal positive control.

### 2.6. Sediment texture analysis

Sediment texture can be categorised through the proportion of sand, silt and clay found within it. Following the collection of the sediment eDNA sample, the remaining homogenised mixture of distilled water and collected sediment was saved. This mixture was allowed to dry completely before the proportions of sand, silt and clay were analysed using a LaMottle Company soil texture test kit following the manufacturer’s instructions [48]. This procedure produced a percentage of each of the components within the sediment for each of the four visits, of which the mean was taken for the analysis. In addition, this allowed the sediment texture to be categorised using the United States Department of Agriculture (USDA) soil texture calculator [49].

### 2.7. Statistical analysis

The concentration of DNA recovered was consistently below the limit of quantification [41,50], and so could not be accurately measured. However, single season occupancy models use repeated observations with detection and non-detection data to estimate the probability of detecting a species [51–53]. Occupancy modelling has been widely used with eDNA [11,54–57] to estimate detection probability, with repeated observations represented by replication of qPCR runs. This process allowed detection probability to be estimated, with each sample representing a “site” and each qPCR run considered an independent observation as in a traditional occupancy analysis. Models were fitted in R version 3.4.1 [58] with package Unmarked version 0.12–2 [59], to identify differences in detection probability. Models were fitted using the occu function, with covariates of detection, but with a constant occupancy (i.e., no covariates fitted for occupancy, only for detectability). Site covariates included in the models were the time of year, the type of samples (aquatic or sediment), study area, the pond sediment texture and the HSI score. The default model selection option within package Unmarked was utilised, ranking models based on Akaike Information Criterion (AIC) and weighted to indicate relative model support. AIC model selection was corroborated using package AICcmodavg version 2.1.1 [60] to generate Bayesian Information Criterion (BIC) to confirm relative model support. Models with strong support were identified having a ΔAIC or ΔBIC ≤ 2 with models with a ΔAIC or ΔBIC of >2 but ≤ 7 were considered to have some support [61,62]. AIC and BIC importance weights for the covariates were generated as measures of covariate importance, by summing the respective weights for each model that contains that covariate [62,63]. Covariates were classed as strongly supported by our models if they were significant in all strongly supported models (ΔAIC≤ 2) and had a cumulative AIC or BIC weight of >0.75 [62]. Covariates were considered to be somewhat supported if they were significant in any of the strongly supported models regardless of cumulative AIC or BIC importance weight [62]. Goodness of fit, using the chi-square statistic and c-hat was performed using package AICcmodavg version 2.1.1 [60], and the mb.gof.test function, with a bootstrap value of 1000, for all somewhat or strongly supported models. The model with greatest support was used with the predict function within the Unmarked package to generate predicted detection probabilities under different covariate combinations.

### 2.8. Ethical assessment

The experimental procedure was approved by the University of Kent, School of Anthropology and Conservation, Research and Ethics Committee. Surveys for great crested newts using traditional methodologies were undertaken following best practice guidelines by experienced surveyors and under licence from Natural England Licence number 2015-16607-CLS-CLS. All eDNA sampling was undertaken from water or sediment and no animals were disturbed. PCR Positive control samples were set up from DNA extracts from a long deceased great crested newt held under licence from Natural England licence number 2015-7591-SCI-SCI-1. Data are available in supporting information S1 Data.

## 3. Results

Using the visual survey methods great crested newts were confirmed from all ponds except the two negative control ponds. This result was corroborated with eDNA samples, with no samples from the two negative control ponds found to be positive.

With the exception of the control ponds, each pond was positive using either sediment or water eDNA samples on at least one occasion. The mean number of qPCR replicates amplifying out of a possible eight for water in spring was 5.67 (standard deviation (SD) = 3.24), which compared to 1.83 (SD = 2.60) for sediment; this increased in the summer to 6.22 (SD = 3.42) for water and 3.28 (SD = 3.34) for sediment. A reduction was seen in autumn, 2.11(SD = 2.70) for water and 1.00 (SD = 1.75) for sediment, reducing further into the winter 0.33 (SD = 0.59) for water and 0.78 (SD = 1.06) for sediment. Only one sample from each sediment and water samples showed signs of inhibition. We constructed models to identify what was influencing the differences in detection probability.

The model with the greatest AIC and BIC support (ΔAIC to the second model = 4.95; ΔBIC to the second model = 2.77; Table 1), for the influences on detection probability included detection based on the season, study area, sediment texture and HSI score, as well as whether the sample was water or sediment. No other models were found to have substantial support (ΔAIC or ΔBIC ≤2), although three additional models were shown to have some support (ΔAIC or ΔBIC ≤7; Table 1).

**Table 1:** Influences on detection probability model selection.

Model		nPars	AIC	ΔAIC	AIC weight	AIC Cumulative weight	BIC	ΔBIC	BIC weight	BIC Cumulative weight	GOF - χ^2^	GOF—p-value	GOF-c-hat
Occupancy	Detection												
Constant	Season, Sample Type, Study Area, Texture, HSI Score	13	827.19	0.00	0.90	0.90	841.37	0.00	0.75	0.75	183.9754	1	0.71
Constant	Season, Texture, Sample Type, HSI Score	11	832.14	4.95	0.075	0.97	844.14	2.77	0.19	0.93	188.8944	1	0.73
Constant	Season, Sample Type, Study Area, Texture	12	834.96	7.77	0.018	0.99	848.05	6.68	0.03	1.00	185.9396	1	0.72
Constant	Season, Texture, Sample Type	10	836.23	9.05	0.0097	1.00	847.14	5.77	0.04	0.97	188.4366	0.998	0.73

Occupancy models with most support based on AIC and BIC criteria and ordered with AIC model selection. The six most supported models through both AIC and BIC as well as all models with a ΔAIC or ΔBIC of <10 presented. All models contain variable detection rates but constant occupancy. Goodness of fit (GOF) χ^2^, P-value and c-hat also shown. nPars represents the number of parameters in the model.

**Fig 1:**
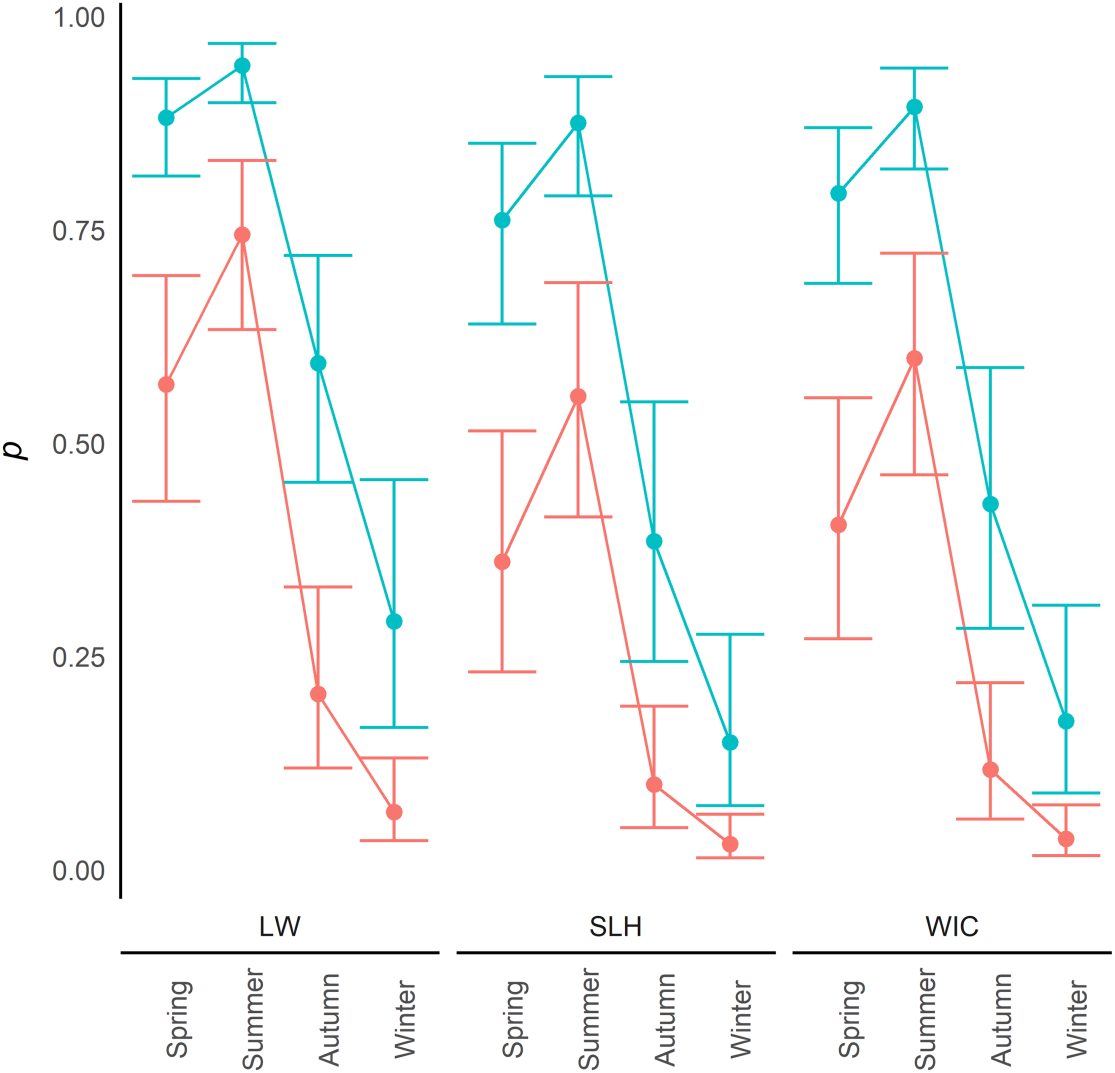
Seasonal detection probability. Variation in detection probability (*p*) between water samples (Blue) and sediment samples (Red) across the seasons, in the different study areas (LW—Little Wittenham; SLH—Stanford-le-Hope; WIC—Wickford), with 95% confidence intervals. These results are based on a clay substrate and an HSI of 0.65 (a score considered mid-range for great crested newt occupancy). Comparisons with ponds in other HSI categories are shown in S1 Fig.

**Fig 2:**
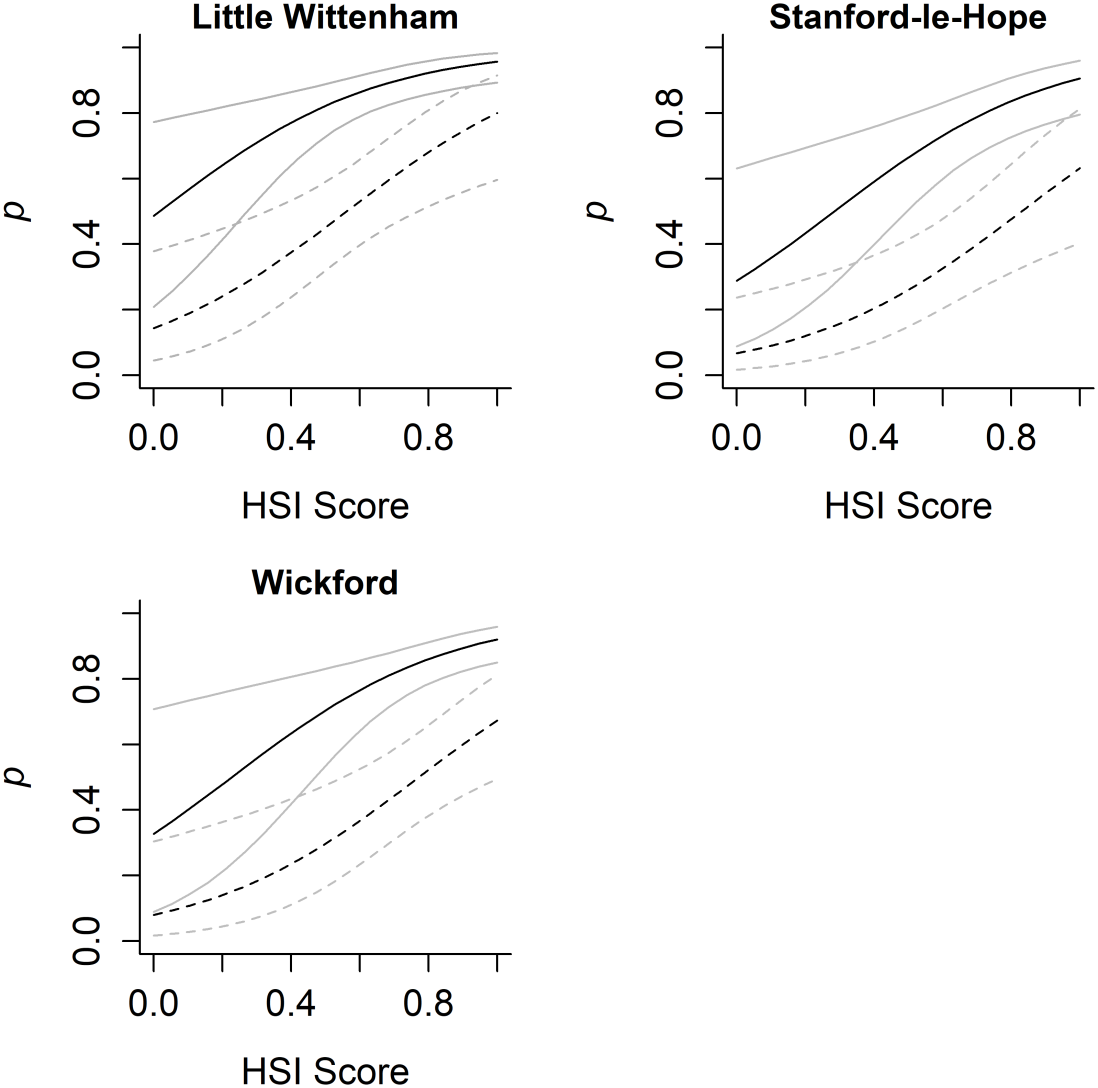
Habitat suitability and detection probability. Variation in detection probability (*p*) between water samples (solid line) and sediment samples (dotted line) in relation to HSI score at three study areas. 95% confidence intervals in light colours. These results are based on a clay substrate and samples collected in spring. Comparisons across the seasons are shown in S2 Fig.

**Fig 3:**
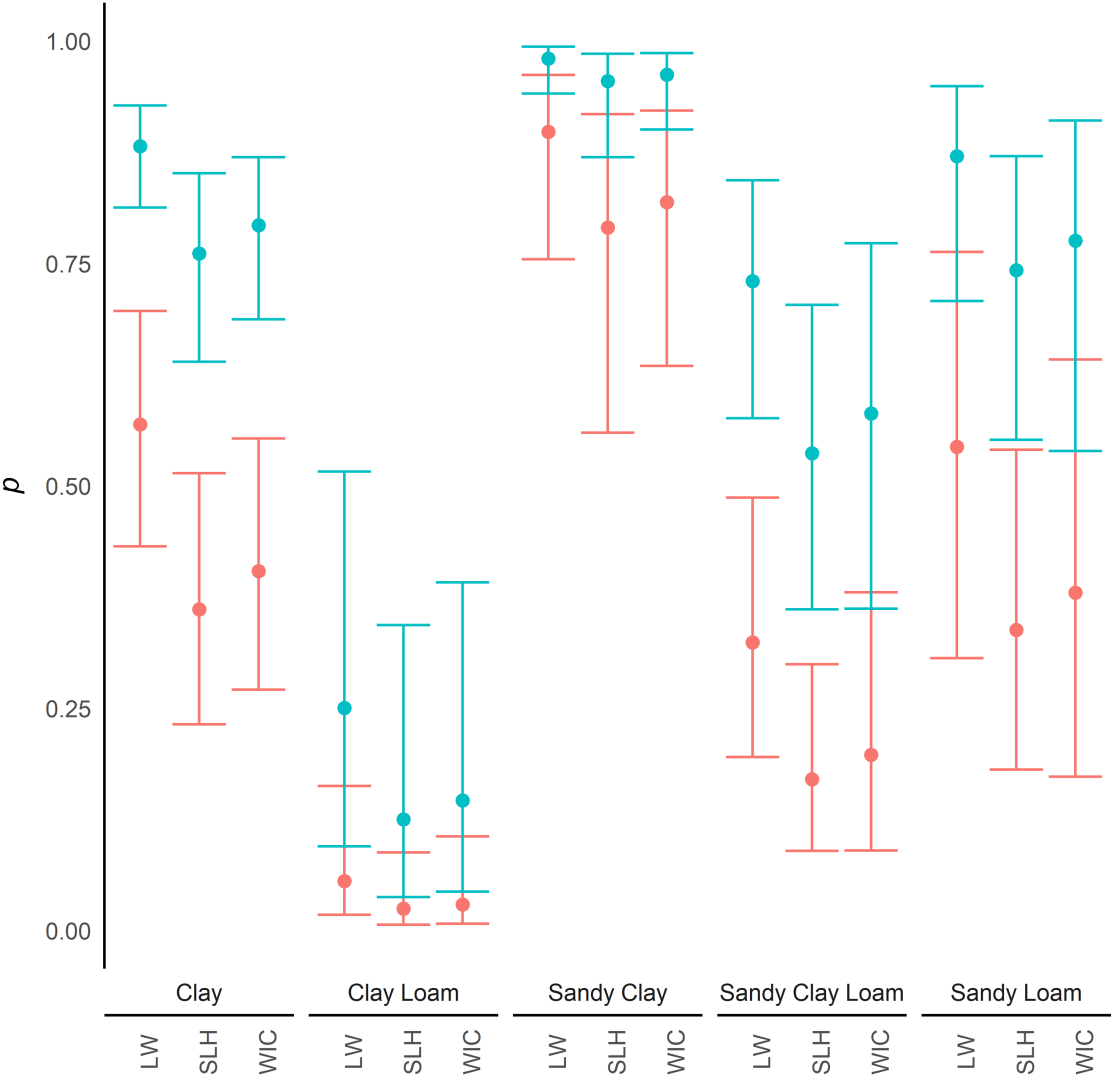
Sediment yype and detection probability. Variation in detection probability (*p*) between water samples (Blue) and sediment samples (Red) with various sediment types, and study area (LW—Little Wittenham; SLH—Stanford-le-Hope; WIC—Wickford), with 95% confidence intervals. These are based on sample collection in spring and an HSI of 0.65 (a score considered mid-range for great crested newt occupancy). Comparisons with ponds in other seasons are shown in S3 Fig.

Within the model of greatest support, samples from water were found to have a significantly greater detection probability of eDNA than samples from sediment (SE = 0.228; z = 7.59; p = <0.0001). Detection of eDNA was significantly increased in samples collected in summer compared to those taken in the spring (SE = 0.264; z = 3.00; p = 0.003), but a significant reduction was seen between spring and autumn (SE = 0.314; z = -5.19; p = <0.0001) as well as between spring and winter (SE = 0.359; z = -8.07; p = <0.0001; Fig 1). Significant differences were also identified between the study areas with Little Wittenham having greater detection probability of eDNA than the two study areas in Essex, Stanford-le-Hope (SE = 0.300; z = -2.83; p = 0.005) and Wickford (SE = 0.327; z = -2.04; p = 0.041). Detection probability was also positively related to the HSI (SE = 1.026; z = 3.09; p = 0.002; Fig 2). eDNA in clay was found to have a significantly greater detection probability than in clay loam (SE = 0.618; z = -5.02; p = <0.0001) and sandy clay loam (SE = 0.341; z = -2.97; p = 0.003). However, eDNA in clay was found to have a lower detection probability than in sandy clay (SE = 0.483; z = 3.93; p = <0.0001), and no significant difference was found between clay and sandy loam substrates (SE = 0.471; z = -0.22; p = 0.828; Fig 3).

Further analysis was undertaken on AIC and BIC importance weights for individual covariates with season (cumulative AIC weight = 1.00; cumulative BIC weight = 1.00), sample type (cumulative AIC weight = 1.00; cumulative BIC weight = 1.00), study area (cumulative AIC weight = 0.92; cumulative BIC weight = 0.78), sediment texture (cumulative AIC weight = 1.00; cumulative BIC weight = 1.00), and HSI score (cumulative AIC weight = 0.98; cumulative BIC weight = 0.94) all strongly supported by the analysis and therefore considered to be important.

## 4. Discussion

The probability of detecting eDNA varies with its concentration [11] and the ability to recover it. We have shown it is possible to detect great crested newts from samples of both pond water and sediment through all seasons, supporting previous work [38]. However, the probability of detecting the target DNA varies through the year in eDNA samples from both water and sediment. We also found that eDNA detection from sediment was lower than from water samples in all seasons. We show that the influences on detection probability vary according to the sediment texture, pond HSI score and the study area.

We demonstrate that detection probability from aquatic samples varies over the year with detection increasing between spring and summer in each of the three study areas, and declining through the autumn to lowest levels in the winter. The trend in the detection probability through the seasons was similar in sediment and aquatic eDNA samples. This finding suggests that contemporary eDNA has a strong influence on detection probability in sediment eDNA samples as seasonal changes in detection exist. However, some detection may be from longer-term DNA deposits within the sediment, as the seasonal changes are not as pronounced as in the water samples. The seasonally variable detection probability, with rates much lower in the winter than spring or summer, suggests a low level of confidence in a negative result outside the core aquatic activity season for the species.

DNA bound to sediment is protected from processes which break it down [13]. During sample collection we were only targeting the very surface of the sediment, which we assume to contain the most recent deposits. Suspended material within the water column including whole cells and extracellular DNA settle out of the suspensions and progressively accumulate within the sediment [5,17], but do not necessarily bind to it. Unbound DNA within sediments has been found to be broken down more quickly than DNA bound to sediments [13]. DNA that has been incorporated into sediments through the settling of cellular material, but remains unbound may explain why our samples did not show a constant level of detection all year. This would suggest unbound target DNA building up through the spring and summer, when the target species is present. However, the amount of target DNA in the sediment is reduced when there are fewer inputs in the autumn and winter.

We also identified that HSI score—a measure of how suitable the habitat is for great crested newts—is positively related to detection. Although all ponds used within the study contained great crested newts, HSI scores ranged from 0.34 to 0.91, with the majority between 0.65 and 0.80. Our data suggests that ponds with higher HSI scores have greater detection probabilities. This may be because the HSI values of ponds in this study were biased towards higher scores. Equally, higher HSI scores and better habitats may mean higher population densities [44,45], and thereby more DNA being released. However, some studies have reported no relationship between HSI value and newt abundance [64] and abundance is not the only influence on eDNA concentration within a pond [41].

Detection of eDNA also varied according to sediment texture. Ponds with clay loam and sandy clay loam had lower detection probability than clay or the other substrate textures. The pattern of lower detectability in clay loam and sandy clay loam was apparent in all four seasons (S3 Fig), but more pronounced in spring and autumn. Fourteen of the nineteen ponds were found to have a clay texture substrate, whereas only two ponds had a sandy clay loam texture, and one of each had sandy loam, sandy clay and clay loam. Due to the unbalanced distribution of the pond substrates between different ponds, other factors that vary between ponds may have exaggerated or masked any influence pond sediment texture had on detection probability. Substrate texture may therefore not be as important as these results suggest although the influence of texture was found to be strong with both the AIC and BIC cumulative weight analysis. As eDNA is often released in particles of sizes large enough to settle into the sediment, which may be within whole cells or aggregations of whole cells, these then accumulate within the sediment but do not necessarily bind to it [16]. The mechanism and capacity for DNA binding would therefore be less important between the sediment textures, and differences between the textures would not be observed.

The sample collection and DNA extraction method allowed for a single homogenous sample to be collected from a pond, rather than multiple independent samples. We chose this method as it allowed for a simple kit-based extraction method with inbuilt steps to remove inhibition. However, most kits designed for extraction of DNA from soil require mechanical cell lysis which have been shown to generate lower yields of eukaryotic eDNA than kits with chemical cell lysis [65–67]. The DNA extraction kit chosen was developed and tested on stool samples which we assumed would have greater efficiency extracting DNA from eukaryotic cells. However, the small volume of sediment used within the analysis may have resulted in low yields and a different extraction process may have recovered more target DNA.

As aquatic eDNA is usually broken down within weeks, detection of great crested newts in water using eDNA indicates current or recent presence of the species [11,47]. Positive detections in the winter therefore suggest some adults or larvae are present in the ponds over this period. Likewise, the seasonal fluctuation of eDNA in sediments also indicates the current or recent presence of the species. Nevertheless, some eDNA within sediment samples may originate from longer-term DNA deposits. However, the lower probability of detection of eDNA extracted from sediments indicates that sediment analysis should not be used to attempt year-round detection of a seasonally aquatic species, at least using the current methods. Refinement of the sample collection protocol, collection of multiple samples from a pond or alterations to the DNA extraction process used may increase DNA recovery rate, detection probability, and ultimately the use of the method for year-round detection of species from sediments.

## Supporting information

S1 Methods Detailed eDNA from sediment extraction protocol. (DOCX)

S1 Data Spreadsheet containing the raw data including, detection/non-detections for qPCR replicates, derived concentrations from qPCR, as well as environmental covariates. (CSV)

S1 Fig Seasonal detection probability. Variation in detection probability (*p*) between water samples (Blue) and sediment samples (Red) across the seasons, in the different study areas, with 95% confidence intervals. Predictions shown assume a clay substrate.
(TIFF)

S2 Fig Habitat suitability and detection probability. Variation in detection probability (*p*) between water samples (solid) and sediment samples (dotted) in relation to HSI score, in all seasons. Little Wittenham (LW), Stanford-le-Hope (SLH), and Wickford (WIC), with 95% confidence intervals. These predictions assume a clay substrate.
(TIFF)

S3 Fig Sediment type and detection probability. Variation in detection probability (*p*) between water samples (Blue) and sediment samples (Red) in relation to sediment types, in the different study areas, and the different seasons, with 95% confidence intervals. All based on an HSI of 0.65 (a score considered mid-range for great crested newt occupancy).
(TIFF)
