# Supplementary material for: Seasonal variation in environmental DNA detection in sediment and water samples
Source: PLoS One. 2018 Jan 19;13(1):e0191737. doi: 10.1371/journal.pone.0191737 (PMC5774844; doi:10.1371/journal.pone.0191737)
Supplement: S1 Methods — (DOCX) [file pone.0191737.s001.docx]

S1 - Methods

## Extraction of sedimentary eDNA samples

Extraction of sedimentary eDNA samples followed modified Qiagen® QIAamp® DNA Stool Mini Kit protocol[44].

1. The 50 mL centrifuge tube containing the ethanol preservative with the suspension of pond sediment and distilled water, was removed from the freezer and shaken vigorously to homogenise the sample. The sample was then centrifuged at 8500 rpm for 30 minutes to separate the sediment from the preservative.
2. The supernatant was carefully poured off and discarded, making sure to leave all sediment within the tube.
3. Sediment was removed from the centrifuge tube and placed on a sterile petri-dish, the sediment was then mixed by hand and 0.5 mL transferred to a 2 mL micro-centrifuge tube. 1.5 mL of ASL buffer from the QIAamp® DNA Stool Mini Kit was added to the micro-centrifuge and mixed by vortexing for 15 seconds.
4. Samples were incubated at 55˚C overnight on a rotating block to separate DNA from the sediment.
5. After incubation the samples were centrifuged at 13,300 rpm for 3 minutes to pellet the unwanted sediment.
6. The supernatant was transferred to a new 2 mL tube and 1 InhibitEX® tablet from the extraction kit added per 1.5mL of sample, the remaining sediment was discarded.
7. The samples with InhibitEX® tables were vortexed for 1 minute and then incubated at room temperature for 1 minute, to allow inhibitors to adsorb onto the InhibitEX® matrix.
8. The samples were then centrifuged at 13,300 rpm for 12 minutes to pellet the tablet and inhibitors. The supernatant was split between two new 2 mL micro-centrifuge tubes. 25 µL of proteinase K was added to each tube, and an equal volume of AL buffer to the supernatant added to each tube.
9. The samples were vortexed for 15 seconds and incubated at 70˚C for 15 minutes.
10. Equal volumes of 100% ice cold ethanol, to the volume of AL buffer added in step eight was then added to each tube, and mixed through vortexing.
11. 600 µL of sample was then transferred to the QIAamp® spin column provided. This was centrifuged at 13,300 rpm for 1 minute, trapping the DNA on the spin column, and flow through discarded.
12. This process was repeated until all of the sample had passed through the spin column.
13. Each spin column was then washed with 500 µL of AW1 buffer and centrifuged for 1 minute at 13,300 rpm.
14. A second wash step using 500 µL of AW2 buffer was undertaken centrifuging for 3 minutes at 13,300 rpm both times. This step was repeated to aid in sample cleaning.
15. The spin column was then transferred to a new 1.5 mL microcentrifuge tube for the elution step and 100 µL of hot AE buffer pipetted directly onto the spin column membrane, this was then incubated at room temperature for 45 minutes before centrifuging at 8,000rpm for 1 minute.
16. A second elution step was undertaken; a further 100 µL of hot AE buffer was added directly to the spin column membrane, and incubated at room temperature for 15 minutes before centrifuging at 8,000rpm for 1 minute.
17. eDNA extracts were stored at -20 ˚C until the qPCR analysis.

## Great crested newt PCR primer and Minor Groove Binding probe

The qPCR primers and probe were developed by Thomsen *et al.* (2012) [45], and amplify an 81 base pair region of the Cytochrome b gene of the mitochondrial genome.

- Forward primer TCCBL – CGTAAACTACGGCTGACTAGTACGAA
- Reverse primer TCCBR – CCGATGTGTATGTAGATGCAAACA
- Minor groove binding probe TCCB.probe - CATCCACGCTAACGGAGCCTCGC

## qPCR assay and conditions

qPCR analysis was run with 8 technical replicates, with standards from diluted great crested newt tissue extracts as positive controls and no template negative controls on each 96-well plate. The quantitative PCR was performed as per Biggs et al. (2015) [39] in a final volume of 25 µL made up of:

- 3 μL of template DNA
- 12.5 μL of TaqMan® Environmental Master Mix 2.0 (Applied Biosystems™)
- 6.5 μL of ddH_2_O
- 2 μL of primer (1 μL each of primer 10 μM TCCBL and TCCBR)
- 1 μL of probe (2.5 μM TCCB Probe)

The PCR was performed under thermal cycling at 50 °C for 5 minutes and 95 °C for 10 minutes, followed by 55 cycles of 95 °C for 30 seconds and 52°C for one minute.
