# Supplementary figures and images for: Seasonal variation in environmental DNA detection in sediment and water samples
Source: PLoS One. 2018 Jan 19;13(1):e0191737. doi: 10.1371/journal.pone.0191737 (PMC5774844; doi:10.1371/journal.pone.0191737)

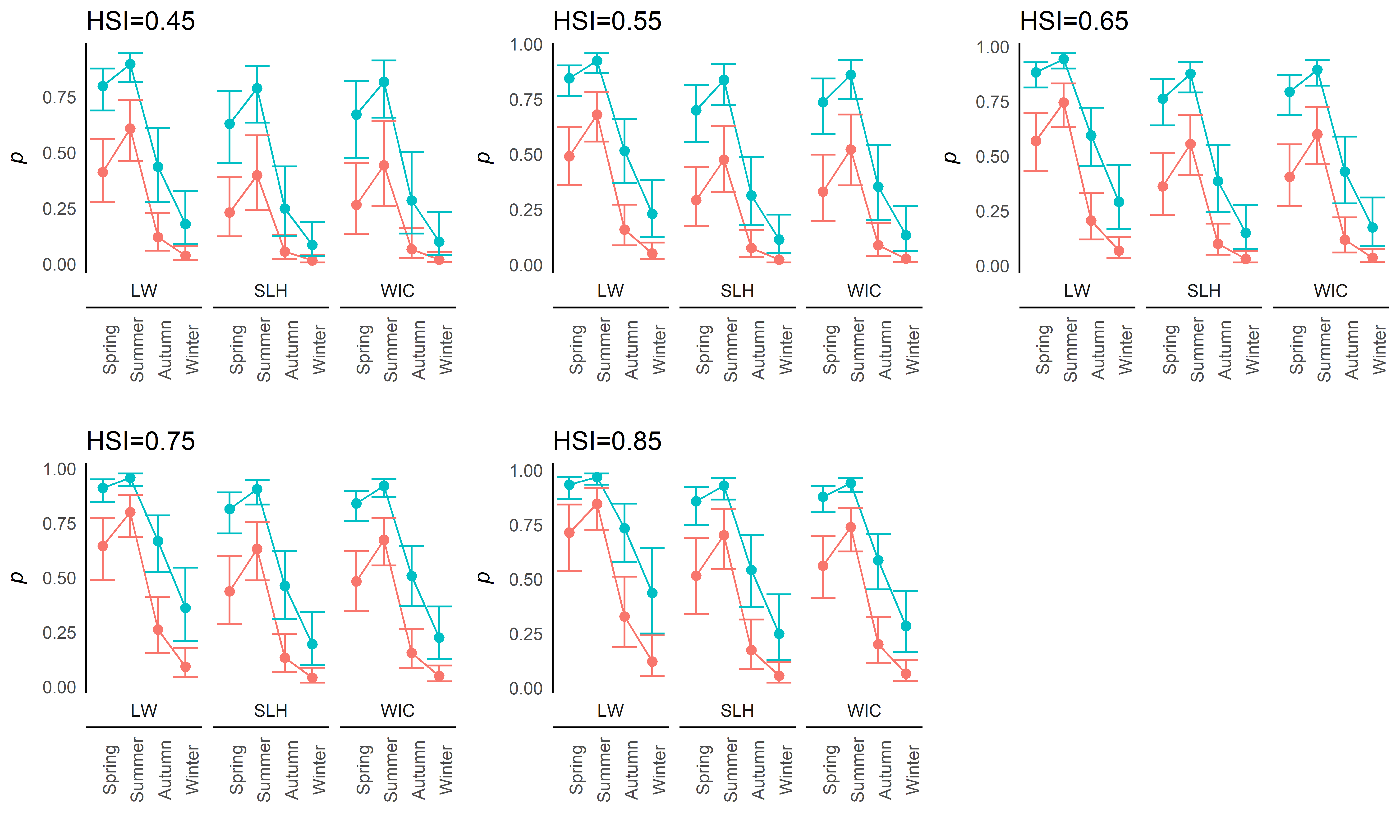

Supplement: S1 Fig — Variation in detection probability (p) between water samples (Blue) and sediment samples (Red) across the seasons, in the different study areas, with 95% confidence intervals. Predictions shown assume a clay substrate. (TIFF) [file pone.0191737.s003.tiff]

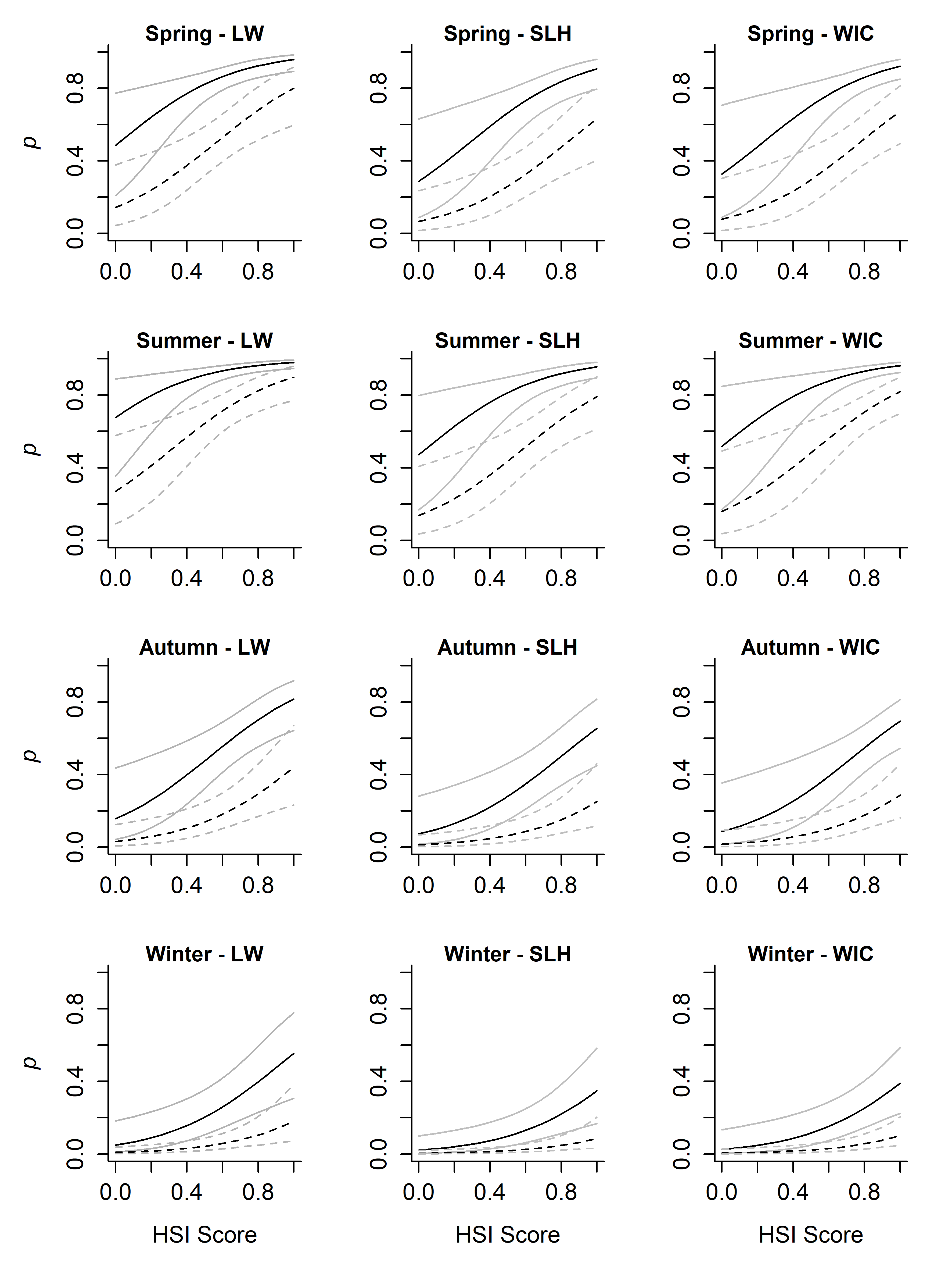

Supplement: S2 Fig — Variation in detection probability (p) between water samples (solid) and sediment samples (dotted) in relation to HSI score, in all seasons. Little Wittenham (LW), Stanford-le-Hope (SLH), and Wickford (WIC), with 95% confidence intervals. These predictions assume a clay substrate. (TIFF) [file pone.0191737.s004.tiff]

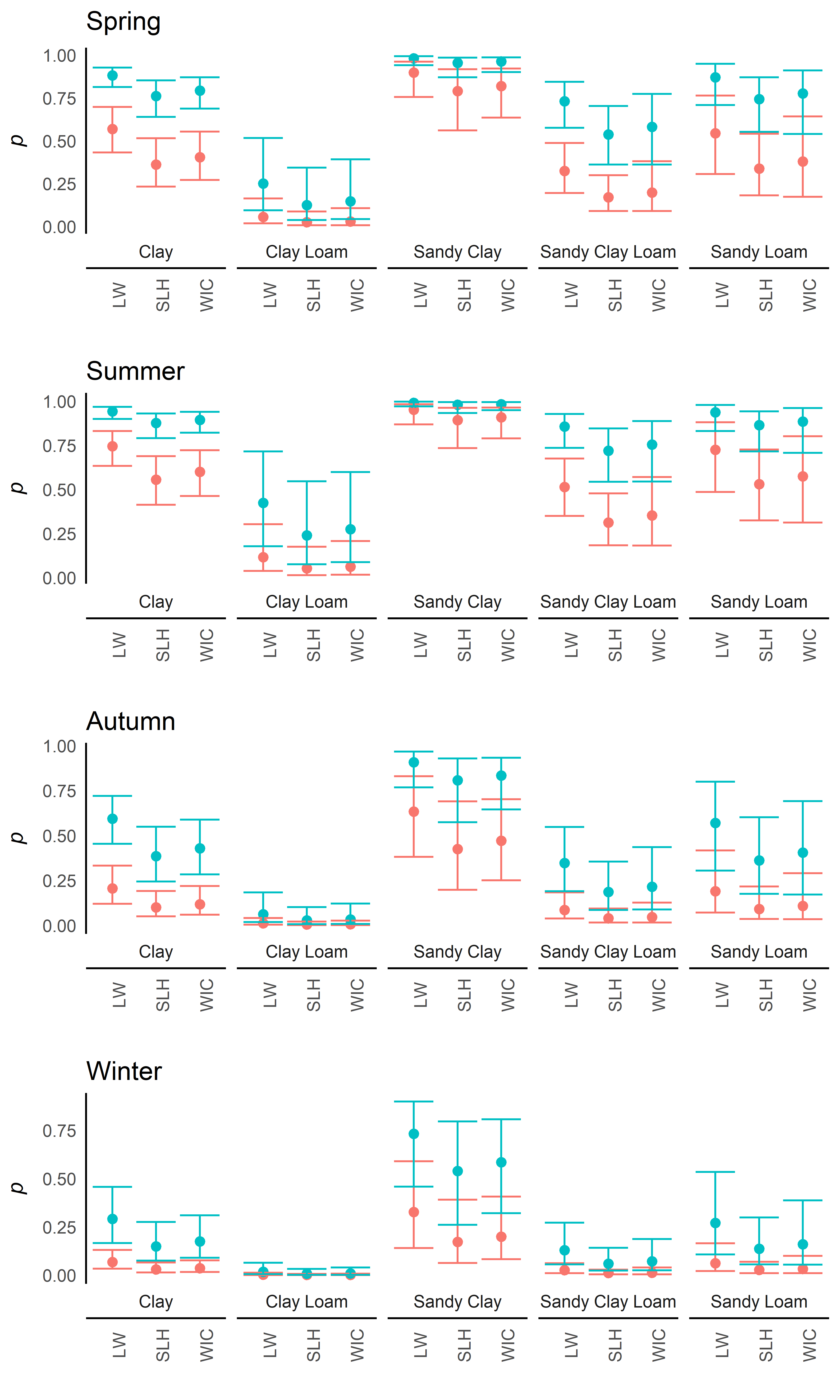

Supplement: S3 Fig — Variation in detection probability (p) between water samples (Blue) and sediment samples (Red) in relation to sediment types, in the different study areas, and the different seasons, with 95% confidence intervals. All based on an HSI of 0.65 (a score considered mid-range for great crested newt occupancy). (TIFF) [file pone.0191737.s005.tiff]
